# Effect of smoking status on immunotherapy for lung cancer: a systematic review and meta-analysis

**DOI:** 10.3389/fonc.2024.1422160

**Published:** 2024-10-08

**Authors:** Dachen Luo, Dongmei Yang, Dan Cao, Zonglian Gong, Fang He, Yaqin Hou, Shan Lin

**Affiliations:** ^1^ Department of Respiratory and Critical Care Medicine, Affiliated Hospital of North Sichuan Medical College, Nanchong, Sichuan, China; ^2^ Department of Pharmacy, Affiliated Hospital of North Sichuan Medical College, Nanchong, Sichuan, China

**Keywords:** lung cancer, smoking status, immune checkpoint inhibitor, systematic review, meta-analysis

## Abstract

**Background:**

Recent studies have yielded conflicting results regarding the relationship between smoking history and the effectiveness of immune checkpoint inhibitors (ICIs) for advanced lung cancer. While some studies have suggested that smoking may enhance the response to immunotherapy in patients with lung cancer, other findings indicate the contrary. Therefore, we conducted a systematic review and meta-analysis to thoroughly examine this association.

**Methods:**

We searched the PubMed, Embase, and Scopus databases for clinical trials comparing immunotherapy with conventional chemotherapy as the primary treatment for advanced lung cancer. A random effects model was used to synthesize hazard ratios (HRs) and 95% confidence intervals (CIs) for overall survival (OS). We also conducted predefined subgroup analyses to investigate the efficacy disparities between never-smokers and smokers who were administered immunotherapy alone or in combination with chemotherapy, as well as the differences between former and current smokers under similar treatment modalities.

**Results:**

Our analysis included data from 17 Phase III clinical trials involving 10,283 patients. The findings indicate that immunotherapy benefits both smokers and never-smokers with lung cancer or non-small cell lung cancer, yielding pooled HRs for OS of 0.74 (95% CI: 0.59–0.92) and 0.73 (95% CI: 0.67–0.80), respectively. A significant interaction effect was not observed (HR: 0.98, 95% CI: 0.77–1.24, p_interaction_ = 0.14), and the tumor type, immunotherapy combination, and type of immunotherapy did not differ among the groups in the subgroup analyses. Similarly, both former and current smokers experienced a significant survival benefit from immunotherapy, with pooled HRs for OS of 0.79 (95% CI: 0.68–0.91) and 0.71 (95% CI: 0.59–0.87), respectively. However, a significant interaction effect was also not observed (HR: 0.91, 95% CI: 0.74–1.11, p_interaction_ = 0.14).

**Conclusion:**

Our findings suggest that smoking status does not affect the effectiveness of immunotherapy for lung cancer treatment. However, additional high-quality clinical trials are needed to confirm this conclusion.

**Systematic review registration:**

https://inplasy.com/register/, identifier INPLASY2023110058.

## Introduction

Immune checkpoint inhibitors (ICIs) are a novel anticancer drug class that targets the immune system by blocking the inhibitory signals between tumor and immune cells, thereby activating and enhancing anti-tumor immune responses. ICIs have shown remarkable clinical efficacy in various malignancies, especially lung cancer, where they have changed treatment strategies and prognoses. The programmed death receptor 1 (PD-1), programmed death receptor ligand 1 (PD-L1), and cytotoxic T lymphocyte-associated protein 4 (CTLA-4) pathways are common mechanisms by which lung cancer cells evade immune surveillance. These pathways lead to immune tolerance and tumor progression by inhibiting the activation and proliferation of anti-tumor T cells. Therefore, using monoclonal antibodies against PD-1/PD-L1 or CTLA-4, either alone or in combination, to relieve the suppression of these pathways is an effective immunotherapy strategy. The United States Food and Drug Administration has approved several drugs, including pembrolizumab and atezolizumab, for the treatment of advanced or metastatic lung cancer. Compared to conventional chemotherapy, these drugs have demonstrated superiority in improving overall survival (OS) and progression-free survival (PFS) in several randomized clinical trials. However, not all lung cancer patients benefit from ICI treatment, and the current response rate remains low, ranging from 10 to 40%, with some toxicity and cost issues (1, 2). Therefore, identifying reliable predictive factors, optimizing treatment regimens, and individualizing treatment plans are current research hotspots and challenges.

Tobacco has a complex chemical mixture that contains more than 60 carcinogens that can cause various cancers (3). Smoking is the main risk factor for lung cancer, and approximately 80% of patients with lung cancer worldwide have a history of smoking. Smoking affects not only the occurrence and development of lung cancer but also the treatment response. Previous studies have found that smoking is related to the treatment response of cancer cells to chemotherapy and radiotherapy. Tobacco smoke can induce normal cells to express PD-L1, allowing them to escape adaptive immunity and promote tumor formation (4). Therefore, the smoking status may also have predictive potential for immunotherapy. Alexandrov et al. found that smoking enhances somatic mutations, thereby increasing the tumor response to anti-PD-1/PD-L1 therapy (5). A subgroup analysis of randomized clinical trials using ICIs as the first-line treatment for advanced non-small cell lung cancer (NSCLC) showed that ICI-treated patients with a history of smoking had significantly longer OS than the control group (6). However, some limitations of these studies may weaken the reliability of their evidence; for example, some studies only recruited a small proportion of non-smokers, limiting the analysis (7, 8). Additionally, previous meta-analyses have shown that smokers, compared to non-smokers, may benefit from immunotherapy, including prolonged OS and PFS; however, these studies only analyzed the effect in patients with NSCLC, and the research is lacking regarding other lung cancer types (3–5).

Considering that smoking status may affect the efficacy of immunotherapy for lung cancer and that this effect could be related to different types of lung cancer, immunotherapy drug types, or immunotherapy drug combinations, we conducted this systematic review and meta-analysis to investigate the relationship between smoking status and immunotherapy outcomes in lung cancer.

## Materials and methods

This systematic review and meta-analysis adhered to the Preferred Reporting Items for Systematic Reviews and Meta-Analyses (PRISMA) reporting guidelines. The study protocol was registered on the Inplasy platform (https://inplasy.com/register/) before data extraction, with the registration number INPLASY2023110058.

### Search strategy

We searched for Phase II and III randomized controlled trials (RCTs) in the PubMed, Embase, and Scopus databases from January 1, 2000, to October 31, 2023. We also reviewed abstracts and presentations from major conference proceedings (i.e., the American Society of Clinical Oncology and the European Society for Medical Oncology) between January 1, 2014, and October 31, 2023. Two researchers (Dachen Luo and Yaqin Hou) independently searched the databases using the following terms: “lung cancer,” “small cell lung cancer,” “SCLC,” “non-small cell lung cancer,” “NSCLC,” “CTLA-4,” “cytotoxic T lymphocyte-associated protein 4,” “PD-1,” “programmed death receptor 1,” “immune checkpoint inhibitors,” “PD-L1,” “programmed death receptor ligand 1,” “immunotherapy,” “ipilimumab,” “tremelimumab,” “nivolumab,” “pembrolizumab,” “serplulimab,” “durvalumab,” “tislelizumab,” “atezolizumab,” “camrelizumab,” “sintilimab,” “toripalimab,” “sugemalimab,” and “penpulimab.” Finally, the reference lists of the included studies were reviewed. **Table 1** in the appendix details the search strategy.

**Table 1:** Characteristics of the including trails.

Study ID	Phase	Tumor type	Treatment groups	Patients	Non-smokers, n (%)	Smokers, n (%)	Median follow-up time (months) ^a^	Median age (years) ^b^	Overall (HR, 95% CI) ^c^	HR (95% CI) for non-smokers ^d^	HR (95% CI) for smokers ^e^	HR (95% CI) for former smokers ^f^	HR (95% CI) for current smokers ^g^
CheckMate-227 (11)	3	NSCLC	PD-L1+CTLA-4	1166	157 (13.5%)	996 (85.4%)	29.3	64 vs. 64	0.73 (0.64-0.84)	0.96 (0.66-1.41)	0.72 (0.62-0.84)	–	–
CASPIAN (14)	3	SCLC	PD-1+ CT	537	37 (6.9%)	500(93.1%)	14.2	62 vs. 63	0.73 (0.59-0.91)	0.90 (0.40-2.11)	0.72 (0.58-0.91)	–	–
IMpower130 (6)	3	NSCLC-A	PD-L1 + CT	679	65 (9.6%)	614(90.4%)	18.5	64 vs. 65	0.79 (0.64-0.98)	0.55 (0.26-1.19)	0.81 (0.65-1.02)	–	–
IMpower110 (15)	3	NSCLC-A	PD-L1+ CT	205	24 (11.7%)	181(88.3%)	15.7	64 vs. 65	0.59 (0.40-0.89)	1.83 (0.63-5.31)	–	0.60 (0.36-1.00)	0.35 (0.14-0.88)
IMpower131 (16)	3	NSCLC-C	PD-L1+ CT	683	55 (8.1%)	627 (91.8%)	18.1	66 vs. 65	0.88 (0.73-1.05)	0.85 (0.43-1.68)	0.87 (0.72-1.05)	–	–
MYSTIC (17)	3	NSCLC	PD-1	325	45 (13.8%)	280 (86.2%)	30.2	64 vs. 64.5	0.76 (0.56-1.02)	0.60 (0.31-1.18)	0.77 (0.58-1.02)	–	–
KEYNOTE-604 (18)	3	SCLC	PD-L1+ CT	453	16 (3.5%)	437(96.5%)	21.6	64 vs. 65	0.80 (0.64-0.98)	–	–	0.71 (0.49-1.02)	0.86 (0.66-1.11)
IMpower132 (19)	3	NSCLC-A	PD-L1 + CT	578	67 (11.6%)	511 (88.4%)	28.4	64 vs. 63	0.86 (0.71-1.06)	0.78 (0.42-1.43)	0.89 (0.72-1.09)	–	–
CheckMate 9LA (20)	3	NSCLC	PD-L1+CTLA-4+ CT	719	98 (13.6%)	621 (86.4%)	13.2	65 vs. 65	0.66 (0.55-0.80)	1.14 (0.66-1.97)	0.62 (0.50-0.75)	–	–
EMPOWER-Lung 3 (21)	3	NSCLC	PD-1+ CT	466	67 (14.4%)	399(85.6%)	16.7	63 vs. 63	0.71 (0.53-0.93)	1.28 (0.53-3.08)	0.61 (0.46-0.82)	–	–
CAPSTONE-1 (22)	3	NSCLC	PD-L1 + CT	462	103 (22.3%)	359(77.7%)	13.5	62 vs. 62	0.72 (0.58-0.90)	0.59 (0.37-0.87)	0.75 (0.59-0.95)	–	–
CHOICE-01 (23)	3	NSCLC	PD-1 + CT	465	145 (31.2%)	320 (68.8%)	16.2	63 vs. 61	0.69 (0.53-0.92)	0.67 (0.39-1.17)	0.70 (0.51-0.98)	–	–
IPSOS (24)	3	NSCLC	PD-L1	453	55 (12.1%)	398(87.9%)	41	75 vs. 75	0.78 (0.63-0.97)	0.70 (0.37-1.35)	–	0.83 (0.64-1.08)	0.65 (0.40-1.07)
ASTRUM-005 (25)	3	NSCLC	PD-1+ CT	585	116 (19.8%)	469(80.2%)	12.3	63 vs. 62	0.63 (0.49-0.82)	0.75 (0.42-1.33)	–	0.59 (0.42-0.83)	0.61 (0.36-1.02)
POSEIDON (26)	3	NSCLC	PD-1 + CT	675	163 (24.1%)	511(75.7%)	34.9	64.5 vs. 64	0.86 (0.72-1.02)	0.92 (0.65-1.31)	–	0.83 (0.66-1.04)	0.77 (0.52-1.12)
POSEIDON (26)	3	NSCLC	PD-1+ CTLA-4 + CT	675	138 (20.4%)	536(79.4%)	34.9	63 vs. 64	0.77 (0.65-0.92)	1.15 (0.79-1.67)	–	0.75 (0.60-0.94)	0.54 (0.37-0.79)
KEYNOTE-026 (27)	3	NSCLC	PD-L1	541	59 (10.9%)	475(87.8%)	13.7	63 vs. 65	1.08 (0.87-1.34)	1.02 (0.54-1.93)	–	1.09 (0.84-1.42)	1.05 (0.63-1.74)
KEYNOTE-189 (28)	3	NSCLC-A	PD-L1	616	73 (11.9%)	543 (88.1%)	10.5	65 vs. 63.5	0.49 (0.38-0.64)	0.23 (0.10-0.54)	0.54 (0.41-0.71)	–	–

Smokers include both former and current smokers.

a represents the median follow-up time in the immunotherapy group.

b represents median age in the immunotherapy group compared with the non-immunotherapy group.

c represents the immunotherapy group compared with the non-immunotherapy group in all populations.

d represents the immunotherapy group compared with the non-immunotherapy group in non-smokers.

e represents immunotherapy group compared with non-immunotherapy group among former smokers.

f represents the immunotherapy group compared to the non-immunotherapy group among current smokers.

NSCLC, non-small-cell lung cancer; NSCLC-A, non-squamous non-small-cell lung cancer; NSCLC-C, squamous non-small-cell lung cancer; CT, chemotherapy; PD-1, programmed cell death protein 1; PD-L1, Programmed cell death 1 ligand 1; CTLA-4, cytotoxic T-lymphocyte-associated protein 4.

### Inclusion and exclusion criteria

The inclusion criteria for the studies were: 1) RCTs; 2) included patients with locally advanced or metastatic lung cancer who were not candidates for surgery; 3) evaluated first-line treatment with ICIs, such as PD-1, PD-L1, and CTLA-4 inhibitors (monotherapy, dual therapy, or combined with chemotherapy), compared with conventional chemotherapy; 4) the full text was available; and 5) reported hazard ratios (HRs) of OS based on the smoking status.

The exclusion criteria were: 1) non-empirical articles, such as reviews, commentaries, and letters to the editor; 2) the use of ICIs as a second-line or later-line treatment; 3) included a control group containing ICIs or anti-angiogenic drugs; 4) no subgroup analysis according to the smoking status; 5) non-English publications; and 6) single-arm Phase I and II trials (i.e., non-randomized trials) and retrospective or prospective cohort studies.

### Data extraction

**Figure 1:**
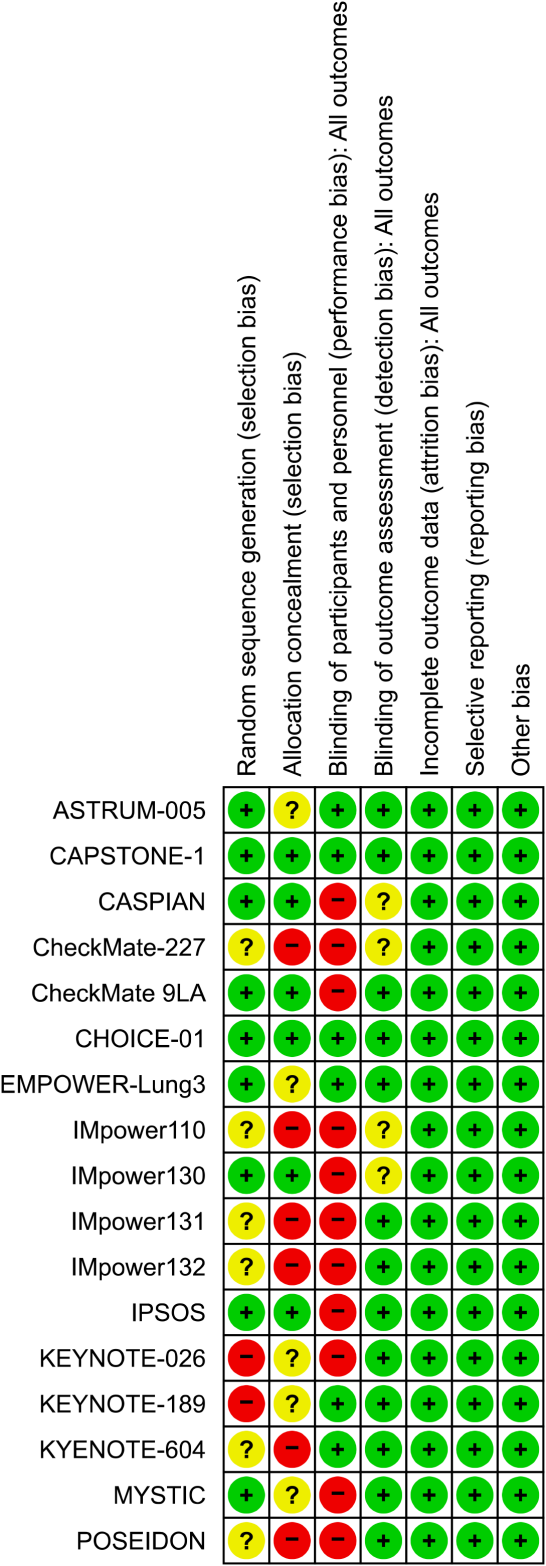
The assessment of risk of bias.

Two authors (Fang He and Dachen Luo) independently extracted the following data from the eligible studies: study name, first author, publication year, study phase, population, study drug, number of patients by smoking status, median follow-up time, median age of the participants (years), and HR of OS based on the smoking status. We managed the literature with Zotero 7.0 (https://www.zotero.org/) and included only the most recent and complete clinical studies. Two authors (Dachen Luo and Dongmei Yang) resolved discrepancies in the literature search and data extraction by consensus. Two authors (Dan Cao and Zonglian Gong) used the Cochrane risk-of-bias tool to assess the risk of bias in the included trials; **Figure 1** presents the risk of bias assessment results.

### Statistical analyses

This study used the Cochrane Risk of Bias Tool to assess the risk of bias in various domains of the included studies, providing a structured method to evaluate research quality with a focus on specific elements that could affect the validity of the results. The domains assessed included selection, performance, detection, attrition, reporting, and other biases. Each domain was rated as having a low, high, or unclear risk of bias, offering a transparent and systematic approach to identifying potential weaknesses within the studies.

The primary outcome was OS after immunotherapy based on the smoking status. We extracted the HRs and 95% confidence intervals (CIs) for OS in smokers and non-smokers in the experimental and control groups from each study and then used a random-effects model to calculate the pooled OS HRs and 95% CIs of smokers and non-smokers separately. We used the Q test to assess the heterogeneity between studies, calculated the I^2^ statistic, used regression and sensitivity analyses to determine the sources of heterogeneity, and performed subgroup analyses based on the regression analysis results to explore the differences in the impact of smoking status on the efficacy of immunotherapy. We only analyzed subgroups that included more than two studies. The effect of smoking status on immunotherapy was compared by interaction analyses. We included only studies corresponding to smokers to ensure the comparisons were comparable in the interaction test for immunotherapy efficacy in non-smokers versus smokers. The chi-square test was used to test the null hypothesis that the interaction between smoking status and immunotherapy efficacy was equal across different subgroups.

All statistical analyses were performed using R (version 4.3.2; R Core Team, Vienna, Austria), and the reported p-values were two-sided, with statistical significance set at p <0.05.

## Results

### Search results and patient characteristics

**Figure 2:**
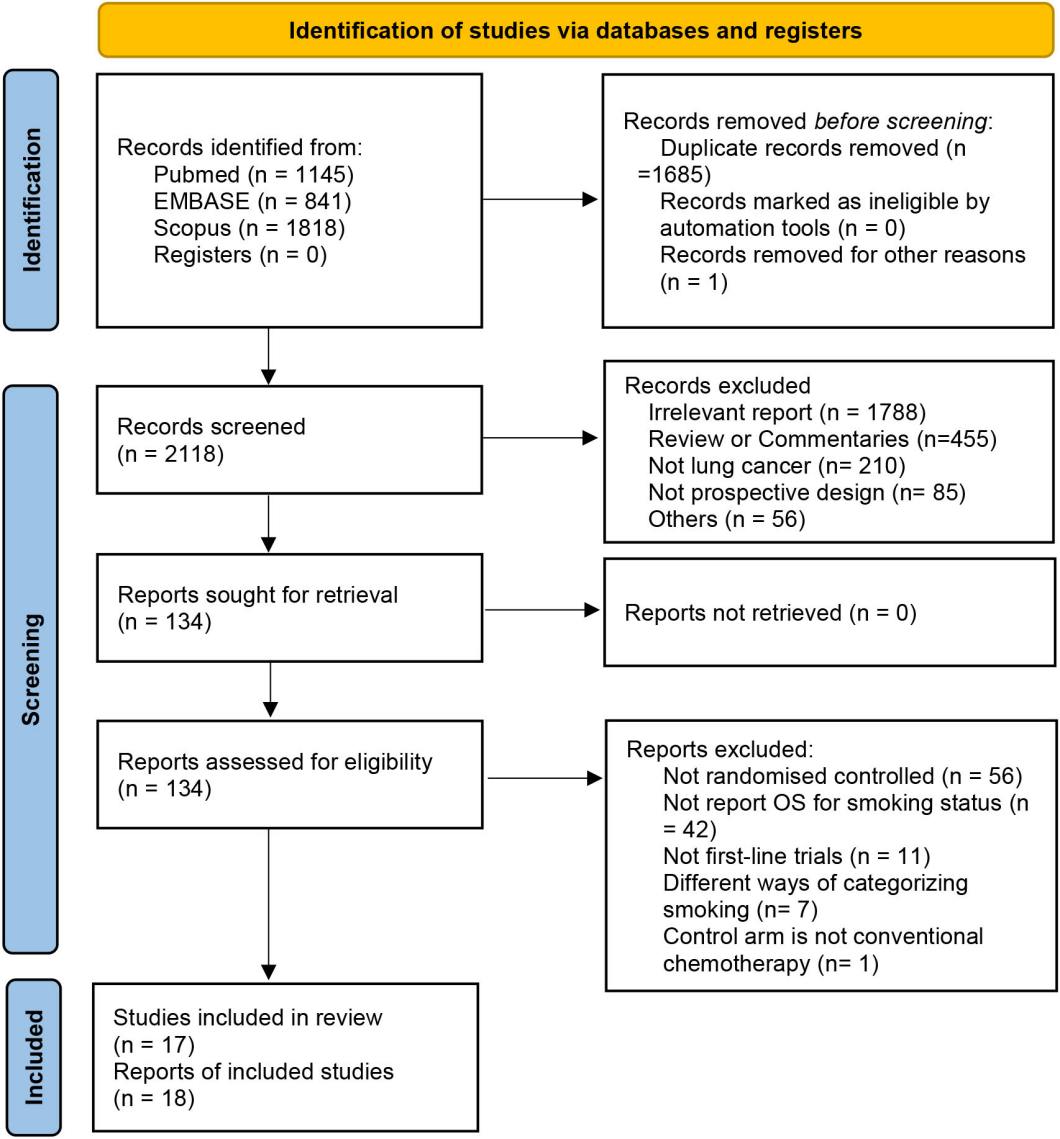
Trial selection flow diagram.

We used a systematic database search strategy to retrieve 3,804 studies that met the criteria from relevant medical literature databases. After deduplication and screening, 2,119 studies were included in the next evaluation. After carefully reviewing the study titles, abstracts, and full texts, we selected 17 clinical trials that met the inclusion and exclusion criteria involving the treatment effects of four combinations of ICIs. Among these, nine studies evaluated PD-L1 monotherapy (n = 4,597), six evaluated PD-1 monotherapy (n = 3,126), and three evaluated PD-1/PD-L1 and CTLA-4 combination therapy (n = 2,560) (**Figure 2**).

All studies were Phase III RCTs involving the first-line treatment effects of four combinations of ICIs for two lung cancer types. Among them, 16 RCTs (n = 9,028) included patients with NSCLC. Based on the histopathological classification, four RCTs included patients with non-squamous carcinoma (n = 2,078), one with squamous carcinoma (n = 683), and 11 with the mixed type (n = 6,532). Two RCTs (n = 990) included patients with small cell lung cancer (SCLC). All included patients had advanced or metastatic tumors, totaling 10,283, of which 12.8% (1,320/10,283) and 85.4% (8,777/10,283) occurred in the never-smoker and smoker groups, respectively. In all studies, the median age ranged from 63 to 75 years, and the median follow-up time ranged from 10.5 to 41 months (**Table 1**).

### Risk of bias assessment

We used the Cochrane risk-of-bias assessment tool to conduct a comprehensive risk-of-bias assessment of the included studies. All studies were rated as low risk for attrition bias, reporting bias, and other biases; most studies were rated as low risk for detection bias, but some were rated as high risk for selection and performance biases (**Figure 1**). Using the Egger’s test, no publication bias was observed in either OS group (p = 0.460).

### Never-smoking patients vs. smoking patients

**Figure 3:**
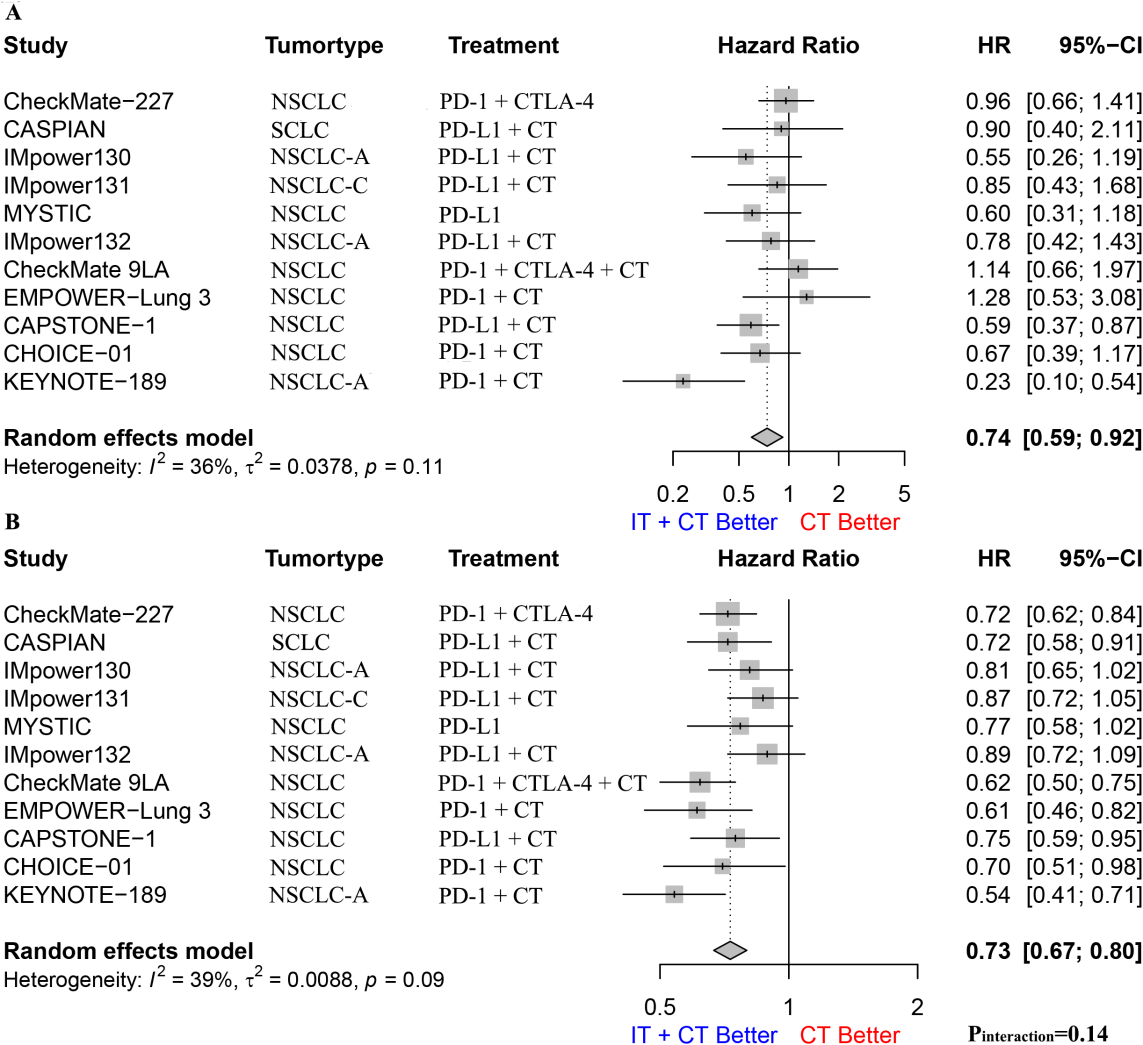
**(A)**. The Hazard ratio for OS between immunotherapy combined with chemotherapy and chemotherapy alone in nonsmoking patients; **(B)**. The Hazard ratio for OS between immunotherapy combined with chemotherapy and chemotherapy alone in smoking patients. The P interaction was calculated from the meta-analyzed HRs of nonsmokers and smokers. NSCLC, non-small-cell lung cancer; NSCLC-A, non-squamous non-small-cell lung cancer; NSCLC-C, squamous non-small-cell lung cancer; CT, chemotherapy; IT, immunotherapy; PD-1, programmed cell death protein 1; PD-L1, Programmed cell death 1 ligand 1; CTLA-4, cytotoxic T-lymphocyte-associated protein 4; OS, overall survival.

We performed a stratified analysis of patients based on their smoking status and found that both smokers and non-smokers benefited from immunotherapy. The pooled OS HR for non-smokers was 0.74 (95% CI: 0.59–0.92; **Figure 3A**), I^2^ was 36%, and p = 0.11. The pooled OS HR for smokers was 0.73 (95% CI: 0.67–0.80; **Figure 3B**), I^2^ was 39%, and p = 0.09. An interaction effect was not observed (HR: 0.98, 95% CI: 0.77–1.24, p_interaction_ = 0.14, **Supplementary Figure S1**).

Due to the scarcity of SCLC studies (one study), we only performed a subgroup analysis for patients with NSCLC, finding that the pooled OS HR for non-smokers was 0.73 (95% CI: 0.58–0.92; **Supplementary Figure S2**), I^2^ was 42%, and p = 0.08. The pooled OS HR for smokers was 0.73 (95% CI: 0.66–0.81; **Supplementary Figure S3**), I^2^ was 45%, and p = 0.06. These results indicate that both smokers and non-smokers benefitted from immunotherapy. However, an interaction effect was not observed (HR: 1.00, 95% CI: 0.77–1.28, p_interaction_ = 0.11, **Supplementary Figure S4**). We also performed subgroup analyses for different types of NSCLC (e.g., squamous carcinoma and adenocarcinoma), different immunotherapy drug combinations (e.g., monotherapy, monotherapy combined with chemotherapy, and dual therapy combined with chemotherapy), and different immunotherapy drug types (e.g., PD-1 and PD-L1); however, we did not find any statistical differences (**Supplementary Figures S5**–**S7**).

### Former smokers vs. current smokers

**Figure 4:**
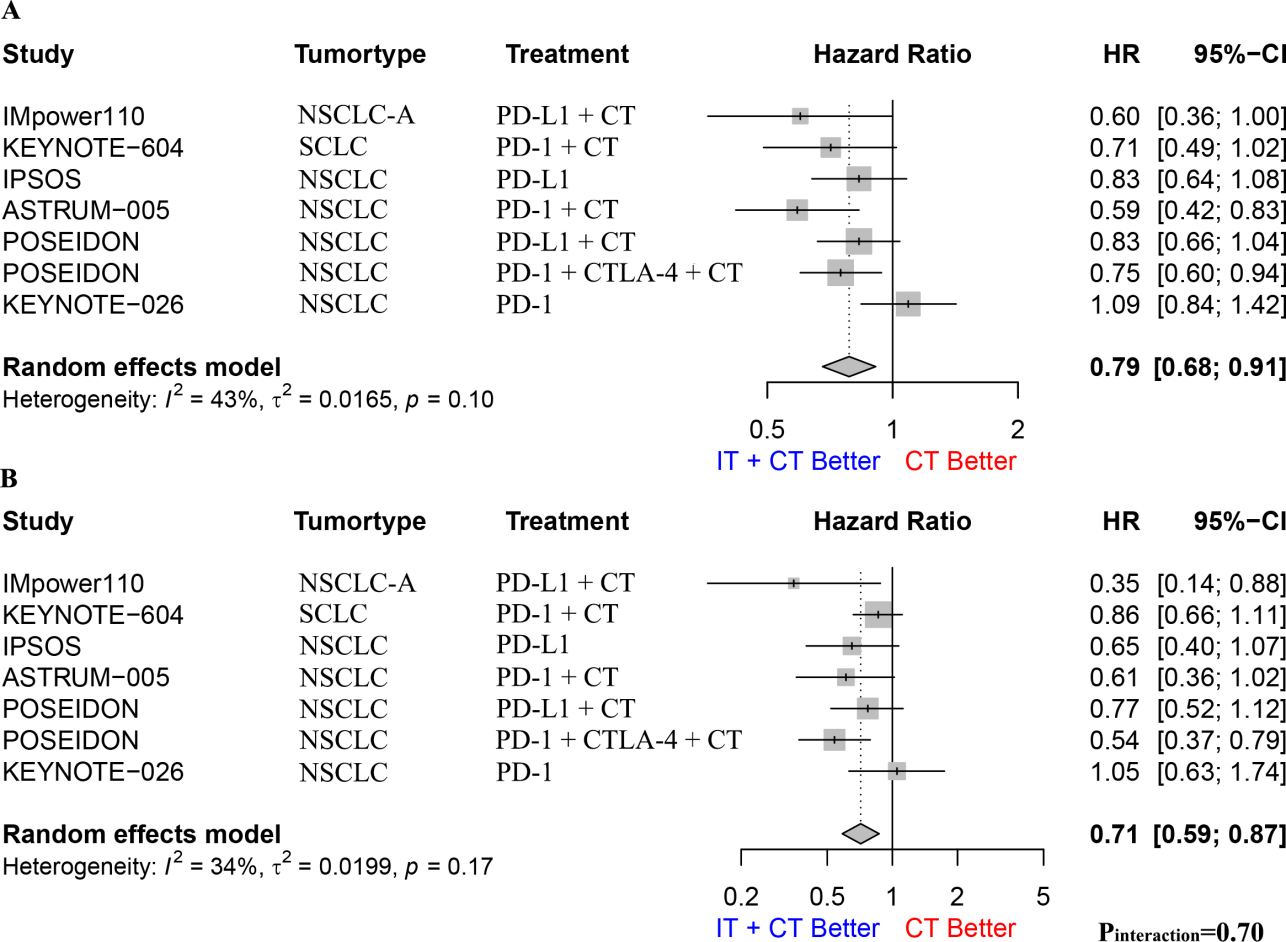
**(A)**. The Hazard ratio for OS between immunotherapy combined with chemotherapy and chemotherapy alone in former smoking patients; **(B)**. The Hazard ratio for OS between immunotherapy combined with chemotherapy and chemotherapy alone in current smoking patients. The P interaction was calculated from the meta-analyzed HRs of former smokers and current smokers. NSCLC, non-small-cell lung cancer; NSCLC-A, non-squamous non-small-cell lung cancer; NSCLC-C, squamous non-small-cell lung cancer; CT, chemotherapy; IT, immunotherapy; PD-1, programmed cell death protein 1; PD-L1, Programmed cell death 1 ligand 1; CTLA-4, cytotoxic T-lymphocyte-associated protein 4; OS, overall survival.

We performed a meta-analysis and interaction effect analysis of patients who formerly smoked and those who currently smoked to evaluate the impact of former and current smoking on the efficacy of immunotherapy. The results showed that both former and current smokers had a survival advantage from immunotherapy, with a pooled OS HR of 0.79 (95% CI: 0.68–0.91, I^2^ = 43%, p = 0.1, **Figure 4A**) for former smokers and 0.71 (95% CI: 0.59–0.87, I^2^ = 34%, p = 0.17, **Figure 4B**) for current smokers. However, the interaction effect analysis did not detect a difference between former and current smokers regarding the efficacy of immunotherapy (HR: 0.91, 95% CI: 0.74–1.11, p_interaction_ = 0.70; **Supplementary Figure S8**). Further subgroup analyses for patients with NSCLC alone yielded similar conclusions (**Supplementary Figure S9**).

## Discussion

Our meta-analysis underscores the critical role of ICIs as frontline therapeutic agents in treating advanced or metastatic lung cancer, revealing their capacity to enhance OS in patients without a smoking status bias. This aligns with previous research findings, reinforcing the consistent efficacy of ICIs. Crucially, our study also delved into the specific impact of former and current smoking on ICI therapy for lung cancer, finding a positive effect on OS across both smoker categories. These insights affirm the broad applicability and potential benefits of ICIs and emphasize their significance in advancing lung cancer treatment paradigms.

The tumors of smokers have a higher mutation and neoantigen load, which increases the ability of the immune system to recognize and eliminate tumor cells, thereby affecting the efficacy of immunotherapy. Using the Cancer Genome Atlas database, Bavarva et al. found that 20% of smokers with NSCLC tumors had at least one mutation in the mucin 4 (*MUC4*), *MUC6*, or *MUC12* genes, whereas only 6% of non-smokers did (7). Alexandrov et al. analyzed the genomic sequences of 5,243 cancers, finding that smoking increased the total number of somatic mutations and was associated with an increased mutational burden of multiple mutation signatures. They also found that one of the signatures was attributed to erroneous replication of DNA damage caused by tobacco carcinogens, whereas the other was attributed to DNA editing by APOBEC cytidine deaminases and endogenous clock-like mutational processes (8). Rizvi et al. further demonstrated that the molecular smoke signature of patients with lung cancer is an important feature of the tumor mutational landscape, which induces higher DNA repair pathway mutations and neoantigen-specific CD T cells, thereby improving the objective response and durable clinical benefit (9). In addition, the tumors of smokers also had higher PD-L1 expression, which may reflect more robust T cell infiltration in the tumor microenvironment, making ICI treatment more effective, as Wang et al. observed that cigarette smoke and the carcinogen benzo[a]pyrene in cigarettes could induce lung epithelial cells to express PD-L1, thereby escaping adaptive immunity and promoting tumorigenesis (10). Therefore, patients with lung cancer who smoke may have better outcomes with immunotherapy than non-smokers. This is consistent with previous studies that found that smokers with lung cancer had better responses to immunotherapy than non-smokers, presenting a conflicting result with our findings (5, 11). The possible reasons for this discrepancy are as follows: 1) Including a smaller number of non-smoking patients may reduce the precision of the combined effect size. For instance, the OS confidence interval for non-smokers is significantly wider compared to smokers. 2) The smoking status of the smoking group was unclear. All included studies merely described the presence or absence of smoking and lacked detailed descriptions of more critical indicators, such as the smoking duration, smoking severity index, and cessation period. It is well known that the incidence and progression of lung cancer are significantly related to the severity of smoking rather than to smoking itself. 3) Although some systematic reviews have found that smoking patients with lung cancer have better post-immunotherapy outcomes than non-smokers, they did not perform an interaction effect analysis, leading to potentially overgeneralized results and undiscovered anomalous heterogeneity.

Smoking affects both the innate and adaptive immune systems of the human body and damage to the innate immune system disappears quickly after smoking cessation; however, damage to the adaptive immune system can last for years. Previous studies (12, 13) found that quitting smoking could significantly increase aryl-hydrocarbon receptor repressor methylation, inhibit aryl hydrocarbon receptor activity, inhibit the proliferation, invasion, migration, and angiogenesis of lung cancer cells, promote tumor cell apoptosis, and reduce immune escape; thus, smoking may enhance the efficacy of immunotherapy for lung cancer patients, and past and current smokers with lung cancer may have different efficacies of immunotherapy. However, both our meta-analysis for lung cancer and previous systematic reviews of NSCLC found that smoking did not improve the efficacy of immunotherapy for lung cancer patients. Simultaneously, this study found that both past and current smokers benefitted from immunotherapy, but the difference between the two groups was statistically insignificant. The possible reasons are as follows: 1) the number of studies included was limited; only seven were included in the analysis, which may have resulted in data bias. 2) The histological types of the study subjects differed and included squamous carcinoma, SCLC, and NSCLC, which may have led to inconsistent results. 3) Past and current smoking data lacked a unified standard, and detailed data on the patient’s smoking status were also missing, such as the smoking duration, smoking severity index, and quitting time.

Our study has some limitations. First, because of the limited number of studies included, we could not perform more detailed analyses of various subgroups, such as those based on the tumor mutational burden, tumor microenvironment, and PD-L1 expression levels. These factors may interact with smoking status and ICI efficacy, which deserves further exploration. Second, since the definition and recording of smoking status varied among different studies, we could not perform accurate stratified analyses of parameters, such as the smoking amount, smoking duration, and quitting time, which may have affected the biological characteristics and immune response of the tumor. Therefore, we suggest that future studies adopt standardized methods to collect and report smoking-related data for more accurate comparisons and evaluations. Finally, this study focused on published and English-language literature; thus, publication bias may exist, as non-English and negative-result publications may have been overlooked. Therefore, our results should be validated and updated in large-scale and long-term follow-up studies.

## Conclusion

Our findings indicate that ICIs significantly enhance OS for patients with lung cancer compared to conventional chemotherapy. Intriguingly, this advantage appeared to be independent of the patient’s smoking status. Although smoking is presumed to be a critical determinant of the efficacy of ICIs, particularly for NSCLC, further rigorous clinical investigations are necessary to substantiate and refine these insights. Moreover, we advocate for future research endeavors to delve deeper into the nuances of how smoking intensity, cessation duration, and other related metrics interact with various prognostic factors in the context of ICI therapy. Understanding the intricate biological underpinnings of how smoking status affects the response to ICIs will be paramount in tailoring more effective and personalized treatment strategies for patients with lung cancer.

## Supplementary material

Supplementary Figure 1 Hazard ratios for the interaction between ICI effect and chemotherapy by non-smoking and smoking.

Supplementary Figure 2 Hazard ratios of OS between immunotherapy and chemotherapy in non-smoking patients with NSCLC.

Supplementary Figure 3 Hazard ratios of OS between immunotherapy and chemotherapy in former-smoke and current-smoke patients with NSCLC.

Supplementary Figure 4 Hazard ratios for the interaction between ICI effect and chemotherapy in NSCLC, by non-smoking and smoking.

Supplementary Figure 5 Hazard ratios of OS between immunotherapy and chemotherapy in smoking non-small cell lung cancer patients by tumor type.

Supplementary Figure 6 Hazard ratios of OS between immunotherapy and chemotherapy in smoking non-small cell lung cancer patients by different combination types of immunotherapy drugs.

Supplementary Figure 7 Hazard ratios of OS between immunotherapy and chemotherapy in smoking non-small cell lung cancer patients by different types of immunotherapy drugs.

Supplementary Figure 8 Hazard ratios for the interaction between ICI effect and chemotherapy by former smoking and current smoking.

Supplementary Figure 9 Hazard ratios for the interaction between ICI effect and chemotherapy in NSCLC, by former smoking and current smoking.

Supplementary Table 1 Search Strategy.
