# Supplementary figures and images for: Effect of smoking status on immunotherapy for lung cancer: a systematic review and meta-analysis
Source: Front Oncol. 2024 Oct 8;14:1422160. doi: 10.3389/fonc.2024.1422160 (PMC11493588; doi:10.3389/fonc.2024.1422160)

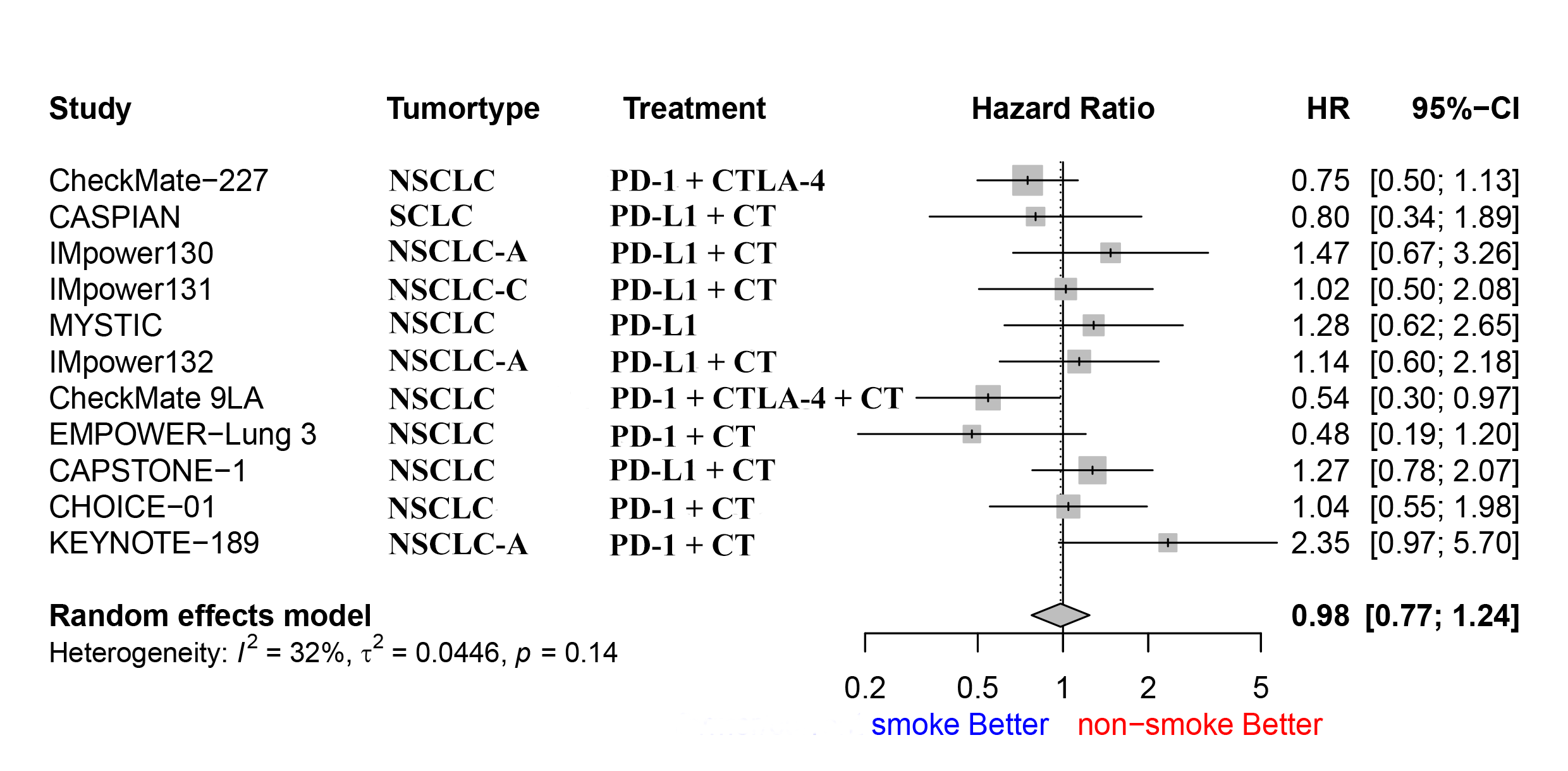

Supplement: Supplementary Figure 1 — Hazard ratios for the interaction between ICI effect and chemotherapy by non-smoking and smoking. [file Image1.tif]

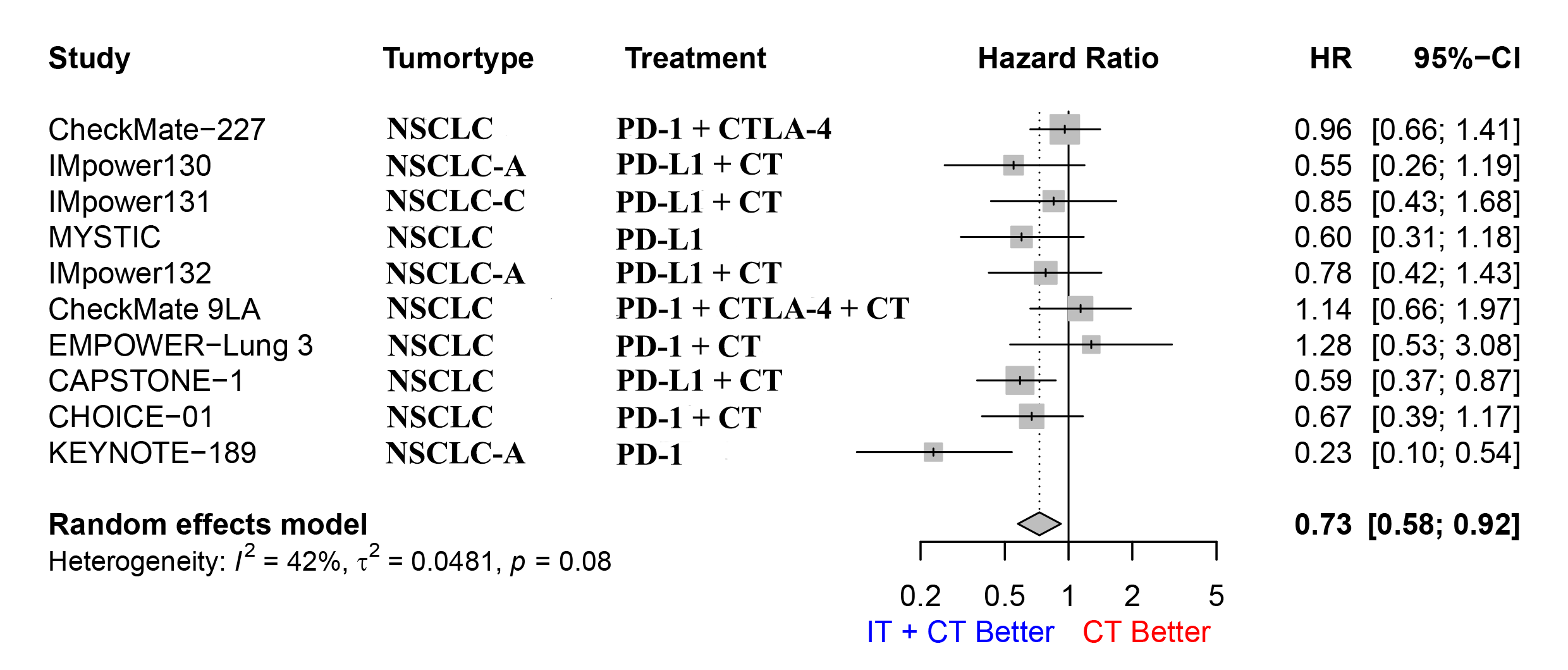

Supplement: Supplementary Figure 2 — Hazard ratios of OS between immunotherapy and chemotherapy in non-smoking patients with NSCLC. [file Image2.tif]

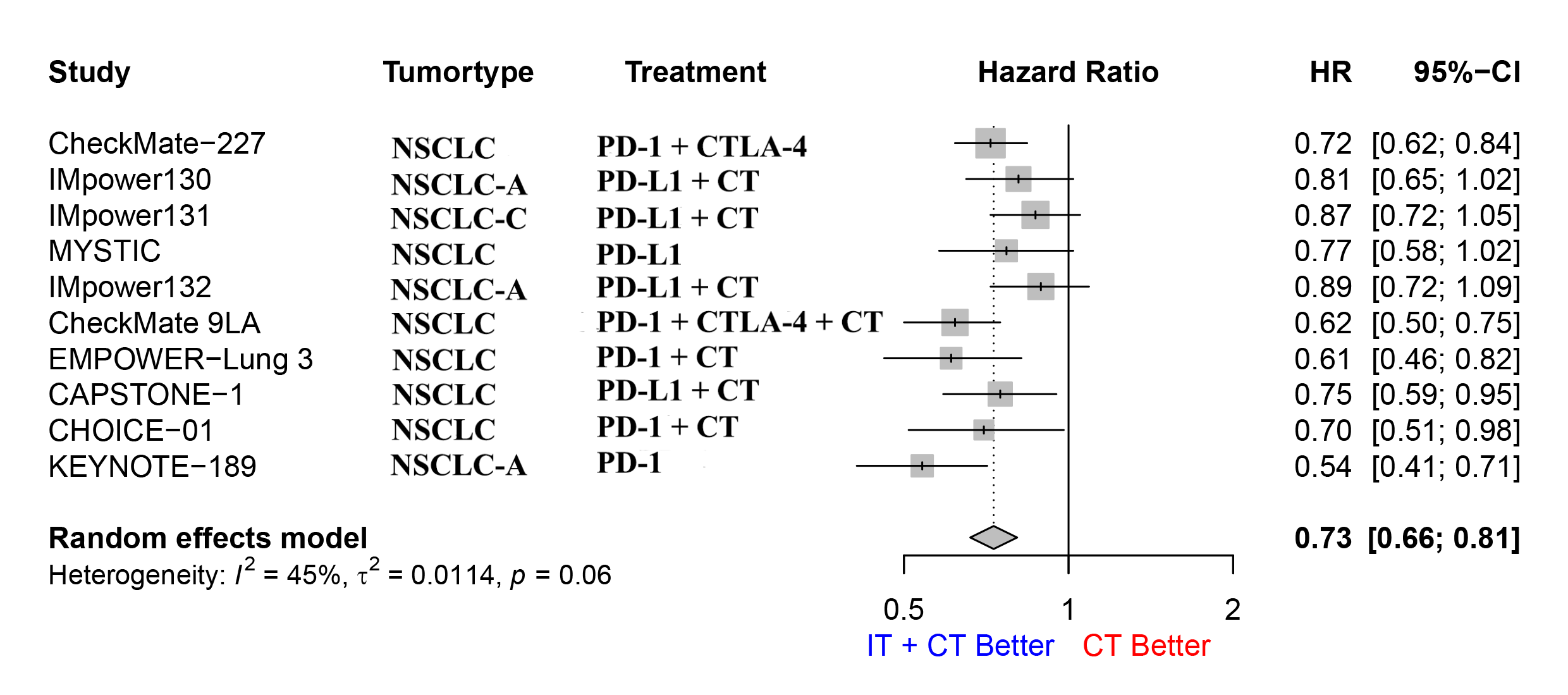

Supplement: Supplementary Figure 3 — Hazard ratios of OS between immunotherapy and chemotherapy in former-smoke and current-smoke patients with NSCLC. [file Image3.tif]

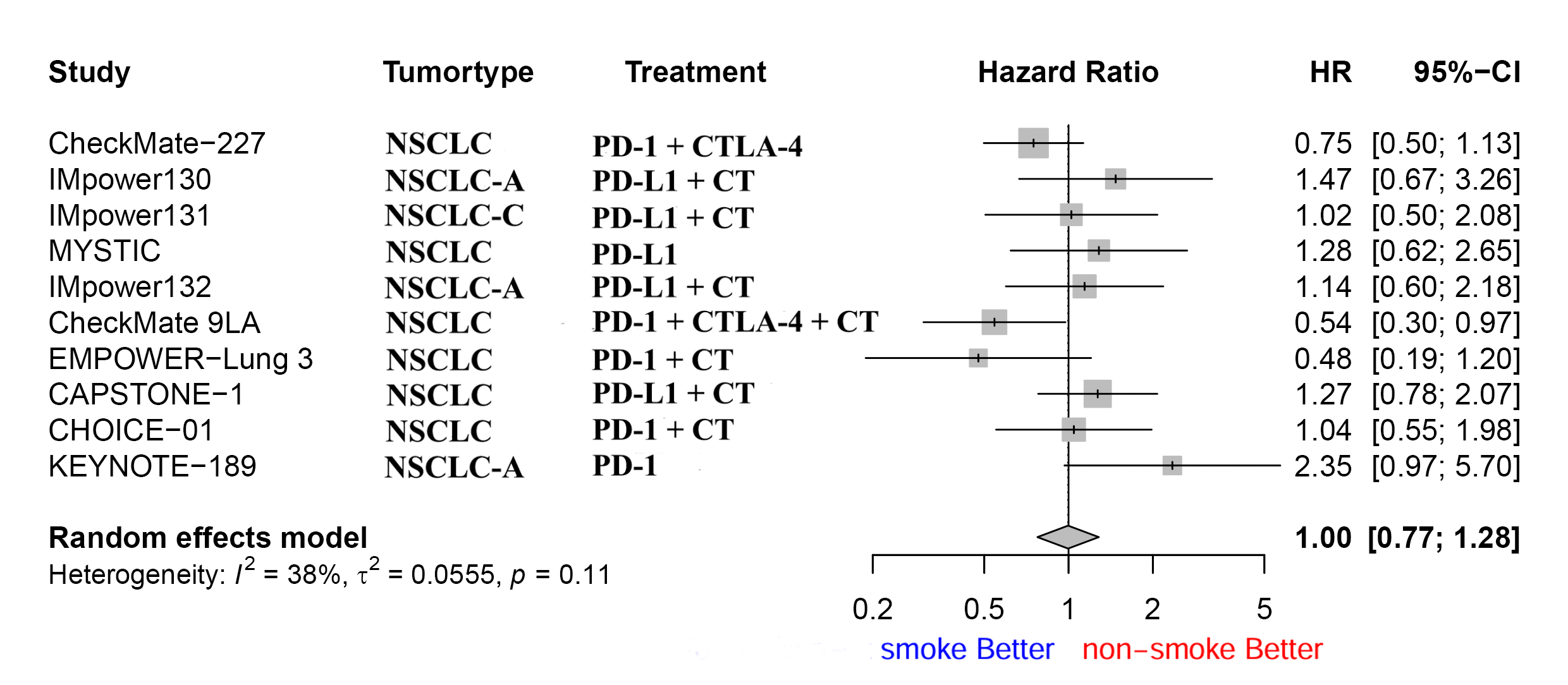

Supplement: Supplementary Figure 4 — Hazard ratios for the interaction between ICI effect and chemotherapy in NSCLC, by non-smoking and smoking. [file Image4.tif]

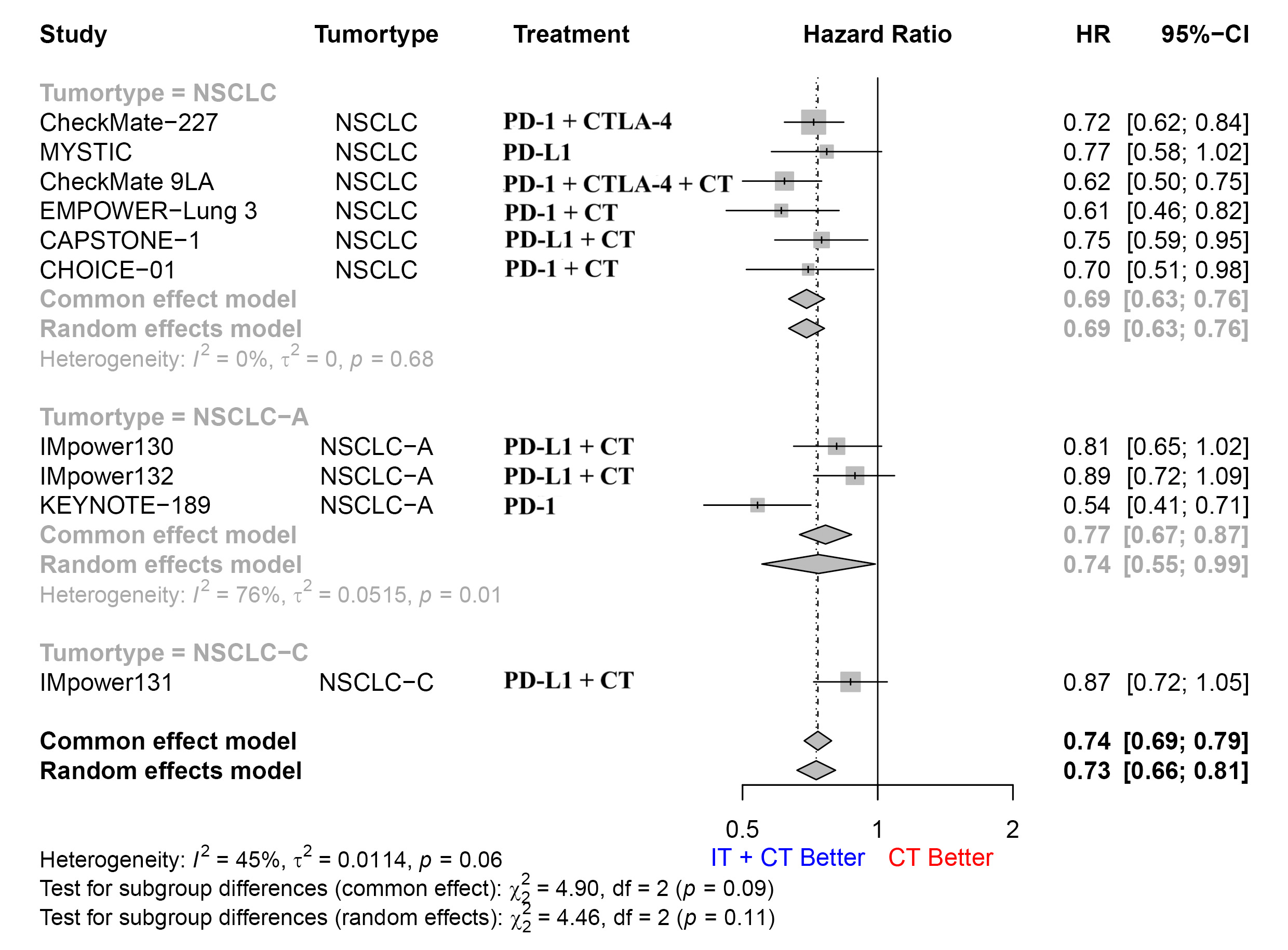

Supplement: Supplementary Figure 5 — Hazard ratios of OS between immunotherapy and chemotherapy in smoking non-small cell lung cancer patients by tumor type. [file Image5.tif]

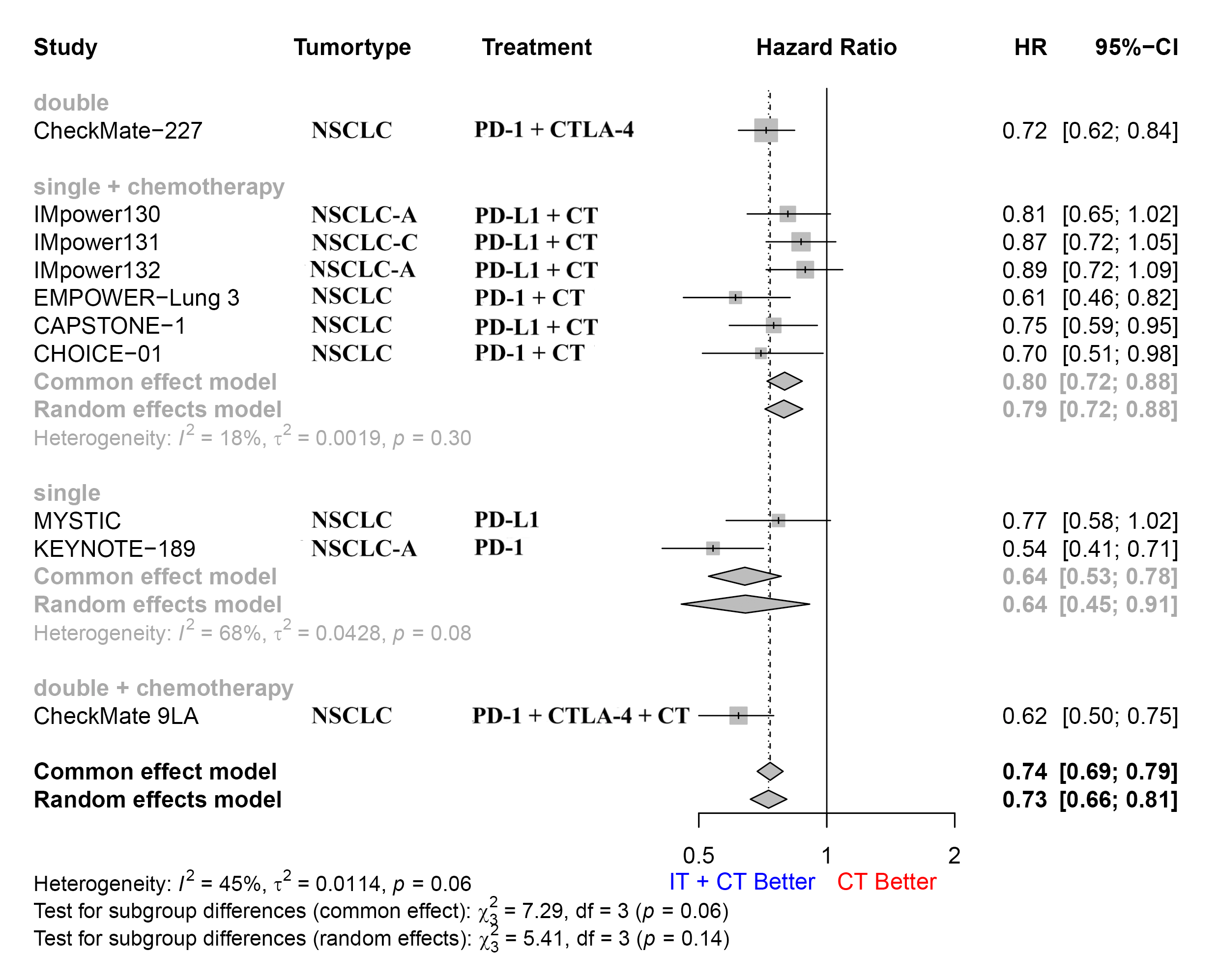

Supplement: Supplementary Figure 6 — Hazard ratios of OS between immunotherapy and chemotherapy in smoking non-small cell lung cancer patients by different combination types of immunotherapy drugs. [file Image6.tif]

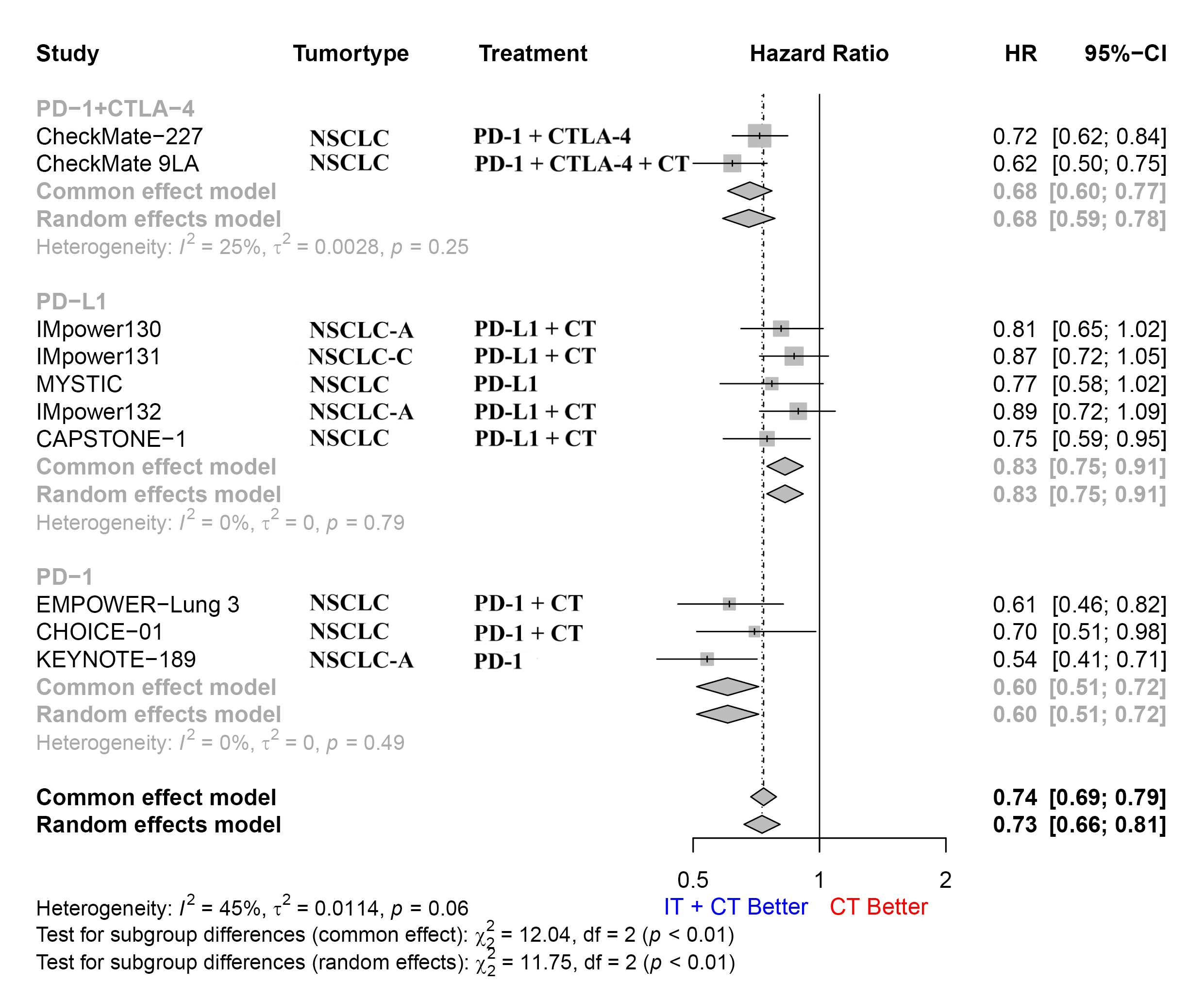

Supplement: Supplementary Figure 7 — Hazard ratios of OS between immunotherapy and chemotherapy in smoking non-small cell lung cancer patients by different types of immunotherapy drugs. [file Image7.tif]

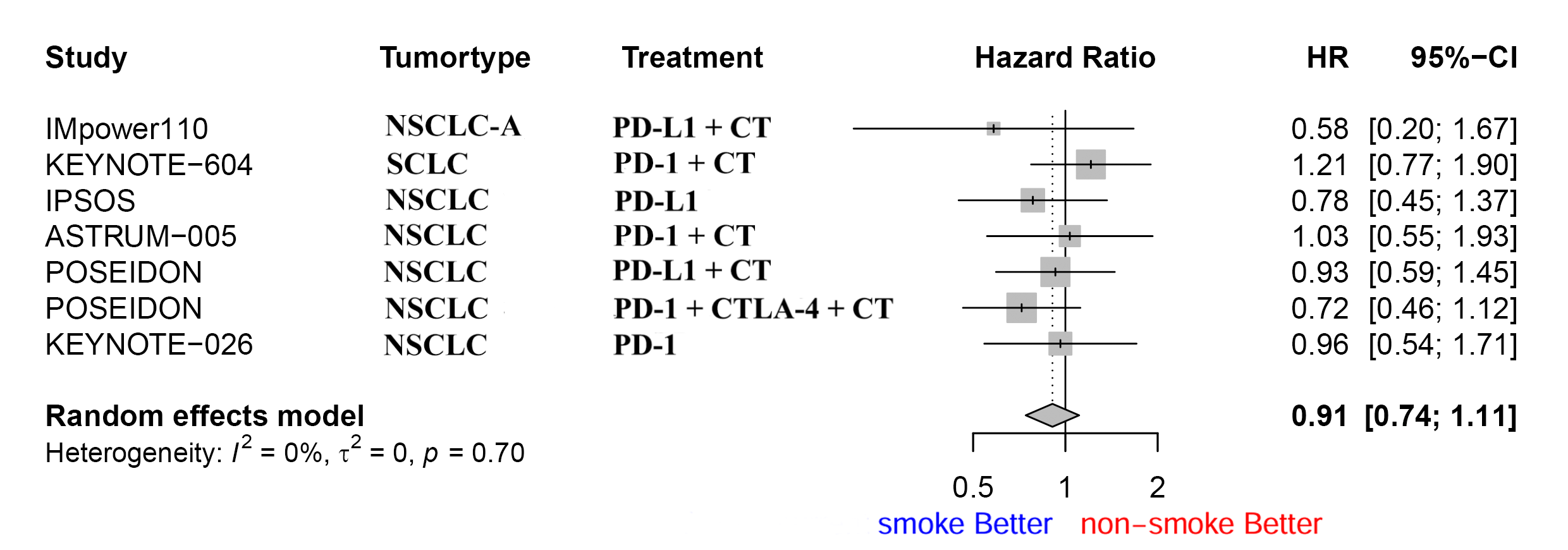

Supplement: Supplementary Figure 8 — Hazard ratios for the interaction between ICI effect and chemotherapy by former smoking and current smoking. [file Image8.tif]

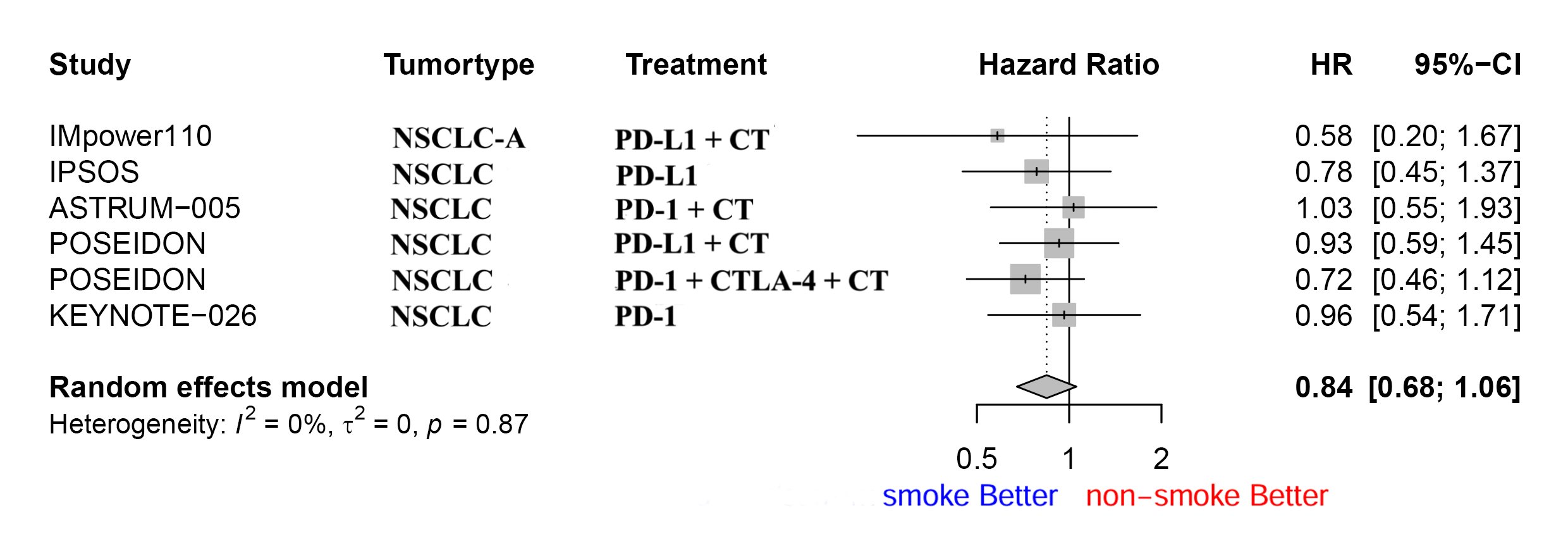

Supplement: Supplementary Figure 9 — Hazard ratios for the interaction between ICI effect and chemotherapy in NSCLC, by former smoking and current smoking. [file Image9.tif]
